# Physiologically based pharmacokinetic modeling of drug–drug interactions between ritonavir‐boosted atazanavir and rifampicin in pregnancy

**DOI:** 10.1002/psp4.13268

**Published:** 2024-11-08

**Authors:** Shakir Atoyebi, Maiara Camotti Montanha, Ritah Nakijoba, Catherine Orrell, Henry Mugerwa, Marco Siccardi, Paolo Denti, Catriona Waitt

**Affiliations:** ^1^ Department of Pharmacology and Therapeutics University of Liverpool Liverpool UK; ^2^ Infectious Diseases Institute Makerere University College of Health Sciences Kampala Uganda; ^3^ Desmond Tutu Health Foundation, Institute of Infectious Disease and Molecular Medicine University of Cape Town Cape Town South Africa; ^4^ Department of Medicine University of Cape Town Cape Town South Africa; ^5^ Joint Clinical Research Centre Kampala Uganda; ^6^ Division of Pharmacology, Department of Medicine University of Cape Town Cape Town South Africa

## Abstract

Ritonavir‐boosted atazanavir (ATV/r) and rifampicin are mainstays of second‐line antiretroviral and multiple anti‐TB regimens, respectively. Rifampicin induces CYP3A4, a major enzyme involved in atazanavir metabolism, causing a drug–drug interaction (DDI) which might be exaggerated in pregnancy. Having demonstrated that increasing the dose of ATV/r from once daily (OD) to twice daily (BD) in non‐pregnant adults can safely overcome this DDI, we developed a pregnancy physiologically based pharmacokinetic (PBPK) model to explore the impact of pregnancy. Predicted pharmacokinetic parameters were validated with separate clinical datasets of ATV/r alone (NCT03923231) and rifampicin alone in pregnant women. The pregnancy model was considered validated when the absolute average fold error (AAFE) for *C*_trough_ and AUC_0‐24_ of both drugs were <2 when comparing predicted vs. observed data. Thereafter, predicted atazanavir *C*_trough_ was compared against its protein‐adjusted IC_90_ (14 ng/mL) when simulating the co‐administration of ATV/r 300/100 mg OD and rifampicin 600 mg OD. Pregnancy was predicted to increase the rifampicin DDI effect on atazanavir. For the dosing regimens of ATV/r 300/100 mg OD, ATV/r 300/200 mg OD, and ATV/r 300/100 mg BD (all with rifampicin 600 mg OD), predicted atazanavir *C*_trough_ was above 14 ng/mL in 29%, 71%, and 100%; and 32%, 73% and 100% of the population in second and third trimesters, respectively. Thus, PBPK modeling suggests ATV/r 300/100 mg BD could maintain antiviral efficacy when co‐administered with rifampicin 600 mg OD in pregnancy. Clinical studies are warranted to confirm safety and efficacy in pregnancy.

**Study Highlights**

**WHAT IS THE CURRENT KNOWLEDGE ON THE TOPIC?**

Ritonavir‐boosted atazanavir co‐administration with rifampicin is not recommended due to limited data on efficacious dosing strategies.

**WHAT QUESTION DID THIS STUDY ADDRESS?**

Dosing strategies for ritonavir‐boosted that could overcome rifampicin‐mediated drug–drug interaction during pregnancy.

**WHAT DOES THIS STUDY ADD TO OUR KNOWLEDGE?**

ATV/r 300/100 mg twice daily can overcome the DDI effect of 600 mg rifampicin in pregnant women.

**HOW MIGHT THIS CHANGE DRUG DISCOVERY, DEVELOPMENT, AND/OR THERAPEUTICS?**

Investigation of dosing strategies that could overcome established drug–drug interactions is important for clinical scenarios with limited drug options.

## INTRODUCTION

Globally, tuberculosis is the leading cause of death among people living with HIV (PLWH).^1^, ^2^ For pregnant women with HIV, tuberculosis is associated with adverse health outcomes for both mothers and their babies, in addition to increased risk of vertical transmission of HIV.^3^ Thus, HIV and TB co‐infection requires adequate treatment of both diseases during pregnancy. Rapid initiation of antiretroviral therapy (ART) during pregnancy helps reduce the risk of vertical transmission of HIV.^1^ Ritonavir‐boosted protease inhibitors (PIs) are recommended for the first‐line ART regimen combination where dolutegravir use is unsuitable,^1^ or for use in second‐line regimens. Recommended PIs, such as atazanavir (ATV), lopinavir (LPV), and darunavir (DRV), are mainly metabolized by CYP3A enzymes^4^, ^5^, ^6^ which are inhibited by ritonavir (RTV).^7^ RTV is also mainly metabolized by CY3A4 with contributions from CY2D6.^7^ Thus, ritonavir‐boosted PIs take advantage of this drug–drug interaction (DDI) with low‐dose RTV to boost the PK of PIs when administered with RTV.^4^, ^5^, ^6^ However, ritonavir‐boosted atazanavir (ATV/r) is preferred by WHO recommendations over ritonavir‐boosted lopinavir (LPV/r) due to its lower pill burden that could improve drug adherence, higher efficacy, and better tolerability.^8^, ^9^

Rifampicin, a well‐known inducer of CYP3A4, is a mainstay of multiple anti‐TB regimens. Co‐administration of rifampicin with boosted atazanavir results in reduced exposure to ATV as with other boosted protease inhibitors.^10^ The 2021 WHO guideline recommends doubling LPV/r standard dose from 400/100 mg twice daily to 800/200 mg twice daily when co‐administered with rifampicin to overcome rifampicin's DDI effect.^1^ However, co‐administration of ATV/r with rifampicin is not recommended by the WHO primarily due to a lack of data on dosing strategies of ATV/r that might safely overcome rifampicin's DDI effect.^1^, ^10^ A recent modeling study, using physiologically based pharmacokinetic (PBPK) models, predicted that increasing the dose of ATV/r 300/100 mg from once daily to twice daily could sufficiently boost ATV concentrations and overcome the DDI effect of rifampicin in non‐pregnant adults.^11^ These results informed the design of a dose escalation trial (DERIVE trial), conducted in PLWH in Uganda by the EDCTP2‐funded VirTUAL consortium. In this trial, ATV/r dosing was escalated from ATV/r 300/100 mg once daily to ATV/r 300/100 mg twice daily in non‐pregnant adults with concomitant administration of standard doses of rifampicin. These results demonstrated that escalation of ATV/r dosing from ATV/r 300/100 mg once daily to ATV/r 300/100 mg twice daily safely overcame the DDI effect of rifampicin in non‐pregnant adults with sufficiently increased ATV concentrations.^8^ Owing to limited data, it is still unclear if similar dosing strategies for ATV/r could also overcome the DDI effect of rifampicin during pregnancy considering the anticipated effects of pregnancy‐induced biological changes on the PK of each drug.^12^, ^13^, ^14^, ^15^ Notably, activities of CYP3A4 and CYP2D6 are also reported to increase during pregnancy which could affect the PK of ATV/r.^6^, ^12^ The aim of this study was to develop and validate a pregnancy PBPK model from the existing adult PBPK model. Subsequently, the pregnancy PBPK model was used to investigate some dosing strategies of ATV/r that might overcome the DDI effect of rifampicin during pregnancy using ATV protein binding‐adjusted 90% inhibitory concentration (PAIC_90_ = 14 ng/mL) as the clinical cut‐off concentration.

## METHODS

### PBPK model description

An existing adult PBPK model with DDI component, described in full by Montanha et al.,^11^ was used for this study. A whole‐body adult PBPK model was used to describe the disposition of atazanavir and ritonavir. For rifampicin, a simple three‐compartment PBPK model was used representing absorption, distribution, and elimination.^11^ Simple illustrations of the model structures for atazanavir, ritonavir, and rifampicin are shown in Figure S1 as earlier reported by Montanha et al.^11^ The model was developed using SimBiology® which is a product of MATLAB R2019a (MathWorks, Natick, US; 2019). Key assumptions include first‐order kinetics, perfusion‐limited drug distribution, no drug reabsorption in the colon, and well‐stirred model distribution. For ATV distribution in the liver, a mechanistic transport‐based model was added which included active efflux by P‐glycoproteins (P‐gp) and uptake‐mediated drug transport mainly mediated by OATP1B.^11^, ^16^ Organ weights were determined by anthropometric equations in literature.^17^ Oral drug absorption and distribution were modeled as earlier described in the non‐pregnant adult PBPK model.^11^

### Intestinal metabolism

Drug clearance in the gut (CL_gut_) by cytochrome P450 3A4 (CYP3A4) without drug–drug interaction (DDI) effect was calculated for ATV and RTV using Equation 1.

(1) $${\mathrm{CL}}_{\mathrm{gut}}={\mathrm{CL}}_{\mathrm{int},\mathrm{CYP}3A4}\times \text{Abundance}$$

where CL_gut_—drug clearance in the gut (L/h), CL_int,CYP3A4_—intrinsic clearance by CYP3A4 (μL/min/pmol), Abundance—abundance of CYP3A4 in the intestine. With the incorporation of the DDI effect, the drug clearance by CYP3A4 in the gut was determined using Equations (2), (3), (4) as previously described.^11^, ^18^

(2) $${\mathrm{CL}}_{\mathrm{gut}}=\frac{{\mathrm{CL}}_{\mathrm{int},\mathrm{CYP}3A4}\times \text{Abundance}\times {\text{Induction}}_{\mathrm{gut}}}{\;{\text{Inhibition}}_{\mathrm{gut}}}$$

(3) $$\;{\text{Induction}}_{\mathrm{gut}}=1+\frac{E_{\max}\times I_{\mathrm{gut}}}{{\mathrm{EC}}_{50}+I_{\mathrm{gut}}}$$

(4) $$\;{\text{Inhibition}}_{\mathrm{gut}}=K_{\text{degradation}}+\frac{\;\;I_{\mathrm{gut}\times K_{\text{inact}}}}{\;I_{\mathrm{gut}+K_{i}}}$$

where CL_gut_—drug clearance in the gut (L/h), CL_int,CYP3A4_—intrinsic clearance by CYP3A4 (μL/min/pmol), Abundance—abundance of CYP3A4 in the intestine, Induction_gut_—induction of CYP3A4 in the gut, Inhibition_gut_—inhibition of CYP3A4 in the gut, *E*_max_—maximum induction, EC_50_—the concentration of the inducer which results in 50% of the maximum induction, *I*_gut_—the concentration of the unbound inducer or inhibitor in the gut, *K*_degradation_—the first‐order rate constant of CYP3A4 degradation, *K*_inact_—the maximal enzyme inactivation rate constant, and *K*_*i*_—the concentration of the inhibitor which results in 50% of the maximum inhibition. CYP3A4 is inducible both by RTV and rifampicin.^11^ Hence, the total induction of CYP3A4 is considered the summation of RTV and rifampicin induction. RTV was modeled as an irreversible inhibitor of CY3A4. In addition, CYP3A4 *k*_degradation_ was set at 0.02/h.^11^, ^19^ In addition, the fraction of the drug escaping the gut was computed using Equation 5.

(5) $$F_{g}=\frac{Q_{\mathrm{gut}}}{Q_{\mathrm{gut}}+\left(f_{u,\mathrm{gut}}\times {\mathrm{CL}}_{\mathrm{gut}}\right)}$$

where *F*_g_—fraction of drug escaping intestinal metabolism, *Q*_gut_—“gut” blood flow (L/h),^20^ *f*_u,gut_–fraction of unbound drug in the gut. The DDI effect in the intestines did not include efflux transporter (P‐gp) interactions.

### Liver metabolism

Similarly, the liver metabolism were modeled as earlier described by Montanha et al.^11^ The total intrinsic clearance (TCL_int,liver_) of ATV was computed using the total intrinsic liver clearance by CYP3A4 (TCL_int,CYP3A,liver_) alone whereas the total intrinsic clearance (TCL_int,liver_) of RTV was the sum of the total intrinsic liver clearance by CYP3A4 (TCL_int,CYP3A4,liver_) and CYP2D6 (TCL_int,CYP2D6,liver_). The total intrinsic liver clearance (TCL_int,liver_) by each enzyme was computed using Equations (6), (7), (8), (9), (10).

(6) $${\mathrm{TCL}}_{\mathrm{int},\mathrm{CYP}3A4,\text{liver}}=\frac{{\mathrm{CL}}_{\mathrm{int},\mathrm{CYP}3A4}\times {\text{Abundance}}_{\mathrm{CYP}3A4}\times \text{MPPGL}\times {\text{Weight}}_{\text{liver}}\times 60\times 1000\times {\text{Induction}}_{\text{liver}}\;}{1,000,000\times {\text{Inhibition}}_{\text{liver}}}$$

(7) $${\mathrm{TCL}}_{\mathrm{int},\mathrm{CYP}2D6,\text{liver}}=\frac{{\mathrm{CL}}_{\mathrm{int},\mathrm{CYP}2D6}\times {\text{Abundance}}_{\mathrm{CYP}2D6}\times \text{MPPGL}\times {\text{Weight}}_{\text{liver}}\times 60\times 1000\;}{1,000,000\;}$$

(8) $$\text{MPPGL}=10^{1.407+\left(0.0158\times \mathrm{Age}\right)-\left(0.00038\times \mathrm{Ag}e^{2}\right)+{\mathrm{Age}}^{3}}$$

(9) $$\;{\text{Induction}}_{\text{liver}}=1+\frac{E_{\max}\times I_{\text{liver}}}{{\mathrm{EC}}_{50}+I_{\text{liver}}}$$

(10) $$\;{\text{Inhibition}}_{\text{liver}}=K_{\text{degradation}}+\frac{\;\;I_{\text{liver}\times K_{\text{inact}}}}{\;I_{\text{liver}+K_{i}}}$$

where TCL_int,CYP,liver_—total intrinsic clearance of drug by a CYP enzyme in the liver (L/h), CL_int,CYP_–intrinsic clearance by the CYP enzymes (μL/min/pmol), Abundance_CYP_–abundance of the CYP enzyme in a milligram of microsomal protein, Weight_liver_—liver weight, Age–age of the individual, MPPGL—milligram of microsomal protein per gram of liver, Induction_liver_—induction of the CYP enzyme in the liver, Inhibition_liver_—inhibition of the CYP enzyme in the liver, *E*_max_—maximum induction, EC_50_—the concentration of the inducer which results in 50% of the maximum induction, *I*_liver_—the concentration of the unbound inducer or inhibitor in the liver, *K*_degradation_—the first‐order rate constant of the CYP enzyme degradation, *K*_inact_—the maximal enzyme inactivation rate constant, and *K*_i_—the concentration of the inhibitor which results in 50% of the maximum inhibition. CYP3A4 *K*_degradation_ in the liver was same as reported for the intestine.

In addition, the ATV transport between the hepatocytes and the systemic circulation by drug transporters was also incorporated into the model as previously described.^11^ Such ATV transport processes included the passive uptake of ATV into hepatocytes, active uptake of ATV into hepatocytes, and the efflux of ATV from the hepatocytes which are computed using Equations (11), (12), (13), respectively. The effect of inducers and inhibitors on the activity of drug transporters facilitating the latter two were also accounted for (Equations 14 and 15).

(11) $$\;{\mathrm{Cl}}_{\text{uptake},\text{passive}}={\mathrm{Cl}}_{\mathrm{int},\mathrm{up},\mathrm{pas}}\times \text{Hepatocytes}\times {\text{Weight}}_{\text{liver}}$$

(12) $$\;{\mathrm{Cl}}_{\text{uptake},\text{active}}={\mathrm{Cl}}_{\mathrm{int},\mathrm{up},\mathrm{act}}\times \text{Hepatocytes}\times {\text{Weight}}_{\text{liver}}\times \frac{\;\;{\text{Induction}}_{\text{uptake}}}{{\text{Inhibition}}_{\text{uptake}}}$$

(13) $$\;{\mathrm{Cl}}_{\text{efflux}}={\mathrm{Cl}}_{\mathrm{int},\text{efflux}}\times \text{Hepatocytes}\times {\text{Weight}}_{\text{liver}}\times \frac{\;\;{\text{Induction}}_{\text{efflux}}}{{\text{Inhibition}}_{\text{efflux}}}$$

(14) $$\;{\text{Induction}}_{\text{uptake}}\;\text{or}\;\;{\text{Induction}}_{\text{efflux}}=1+\frac{E_{\max}\times I_{\text{liver}}}{{\mathrm{EC}}_{50}+I_{\text{liver}}}$$

(15) $$\;{\text{Inhibition}}_{\text{uptake}}\;\text{or}\;\;{\text{Inhibition}}_{\text{efflux}}=1+\frac{\;\;I_{\text{liver}}}{K_{i}}$$

where Cl_uptake,passive_—clearance by passive uptake into hepatocytes, Cl_int,up,pas_—intrinsic passive uptake clearance (μL/min/million hepatocytes), Cl_uptake,active_—clearance by active uptake into hepatocytes, Cl_int,up,act_—intrinsic active uptake clearance (μL/min/million hepatocytes), Cl_efflux_—clearance by efflux from the hepatocytes, Cl_int,efflux_—intrinsic efflux clearance (μL/min/million hepatocytes), Hepatocytes—number of hepatocytes per gram of liver, Weight_liver_—weight of the liver, Induction_uptake_—induction effect on the uptake transporter, Inhibition_uptake_—inhibition effect on the uptake transporter, Induction_efflux_—induction effect on the efflux transporter, Inhibition_efflux_—inhibition effect on efflux transporter, *E*_max_—maximum induction, EC_50_—the concentration of the inducer which results in 50% of the maximum induction, *I*_liver_—the concentration of the unbound inducer or inhibitor in the liver, and *K*_*i*_—the concentration of the inhibitor which results in 50% of the maximum inhibition.

The fraction of the drug escaping first‐pass hepatic clearance (*F*_*h*_) was computed using Equation 16. With the absence of renal clearance contributions in the model, the systemic clearance was computed using Equation 17.^21^

(16) Fh=QhvQhv+fuB:P×TCLint,liver

(17) CLsyst=Qhv×fuB:P×TCLint,liverQhv+fuB:P×TCLint,liver

where *Q*_hv_—rate of blood flow to the hepatic vein (L/h), fu—fraction of unbound drug in the plasma, B:P—blood‐to‐plasma drug partition coefficient, TCL_int,liver_—total intrinsic liver clearance of the drug, CL_hep_—hepatic drug clearance (L/h), *F*_h_—fraction of drug escaping first‐pass metabolism, and CL_syst_—systemic drug clearance (L/h).

The drug‐specific parameters implemented in the model are shown is Tables S1–S3.

### Pregnancy PBPK model development

The validated adult PBPK model for ATV and RTV was feminized by ensuring that all gender‐specific characteristics (e.g., organ weights) were limited to female values only.^17^ Key pregnancy‐induced changes in anatomy, physiology, and metabolic function that are known to affect PK were also incorporated to develop the pregnancy PBPK model.^12^, ^22^, ^23^ These pregnancy‐induced changes are shown in Table 1.

**TABLE 1:** Key pregnancy‐induced biological changes implemented in the pregnancy ATV/r PBPK model.

Parameter	Equation or value for parameter in pregnancy	Reference
Body weight (kg)	$\text{Body weigh}t_{\text{preg}}=\left(\frac{61.1+0.2409\mathrm{GA}+0.0038{\mathrm{GA}}^{2}}{61.1}\right)\times \text{Body weight}$	[12]
Cardiac output (L/h)	$\text{Cardiac outpu}t_{\text{preg}}=301+5.916\mathrm{GA}-0.088{\mathrm{GA}}^{2}$	[12]
Plasma protein concentration (g/L)	$\text{Plasma protein}s_{\text{preg}}=69.7+0.2085\mathrm{GA}-0.0305{\mathrm{GA}}^{2}+0.0006{\mathrm{GA}}^{3}$	[12]
CYP3A4 activity	$\mathrm{CYP}3A4\;\text{activit}y_{\text{preg}}=\left(1+0.0129\mathrm{GA}+0.0005{\mathrm{GA}}^{2}\right)\times \mathrm{CYP}3A4\;\text{activity}$	[23]
CYP2D6 activity	$\mathrm{CYP}2D6\;\text{activit}y_{\text{preg}}=\left(1-0.0163\mathrm{GA}+0.0009{\mathrm{GA}}^{2}\right)\times \mathrm{CYP}2D6\;\text{activity}$	[23]
RIF CL_syst_ (L/h)	${\mathrm{CL}}_{\text{syst},\text{preg}}={\mathrm{CL}}_{\text{syst}}\times 0.86$	[15]

Abbreviations: CL_syst_, apparent clearance; CYP, cytochrome P450; GA, gestational age; Preg, pregnancy; RIF, rifampicin.

### Pregnancy PBPK model validation with clinical PK data during pregnancy

Clinical data used to validate the model included PK data of pregnant women receiving ATV/r 300/100 mg once daily during the second and third trimesters of pregnancy.^13^, ^14^ In addition, sparse clinical data of pregnant women receiving ATV/r 300/100 mg from the VirTUAL clinical trial was also used to validate the model predictions for ATV PK (NCT03923231). Lastly, clinical PK data of pregnant women receiving rifampicin 600 mg once daily was used to validate the model for rifampicin PK.^15^ Some characteristics of the clinical PK data in pregnant women that were used to validate the pregnancy PBPK model for the PK of ATV/r alone and PK of rifampicin alone during pregnancy are described in Table 2.

**TABLE 2:** Characteristics of clinical PK data in pregnancy for ATV/r and rifampicin used for the pregnancy PBPK model validation.

Drug	Dosing regimen	Number of study participants	Time of PK sampling	Reference
ATV/r	Oral ATV/r 300/100 mg repeated once daily	9	Steady‐state during second trimester	[13]
ATV/r	Oral ATV/r 300/100 mg repeated once daily	20	Steady‐state during third trimester	[13]
ATV/r	Oral ATV/r 400/100 mg repeated once daily	20	Steady‐state during third trimester	[13]
ATV/r	Oral ATV/r 300/100 mg repeated once daily	18	Steady‐state during third trimester	[14]
ATV/r	Oral ATV/r 300/100 mg repeated once daily	6	Pooled sparse data collected at steady‐state during second trimester	(NCT03923231)
ATV/r	Oral ATV/r 300/100 mg repeated once daily	6	Pooled sparse data collected at steady‐state during third trimester	(NCT03923231)
Rifampicin	Oral 600 mg repeated once daily	33	Steady‐state	[15]

Abbreviations: ATV/r, ritonavir‐boosted atazanavir; PK, pharmacokinetics.

Model validation was conducted as advised by the European Medicines Agency (EMA) guidelines, that is, the summary statistics (mean or median) of the simulated PK parameters were compared against their corresponding observed PK parameters in relevant clinical studies.^24^ For this study, the model was considered validated when the simulated PK parameters were less than twofold of the observed clinical values. Thus, the absolute average fold error (AAFE), shown in Equation 18, should also be <2.

(18) $$\text{AAFE}=10^{\left|\frac{\sum \log\frac{\text{predicted}}{\text{Observed}}}{N}\right|}$$

where AAFE—absolute average fold error, *N*—number of data points compared, predicted—model predicted value(s), and observed—observed clinical value(s).

The PK parameters evaluated were the area under the plasma drug concentration (AUC), maximum plasma drug concentration (*C*_max_) and the minimum plasma drug concentration (*C*_min_) within a specified time interval.

### PK predictions of ATV/r and rifampicin co‐administration during pregnancy

A virtual population of 100 pregnant women was generated for each simulation. The age range and body weight range of the virtual pregnant women were 25–31 years and 50–117 kg, respectively. The co‐administration of the standard regimens of ATV/r and rifampicin were simulated in virtual pregnant women during second and third trimester of pregnancy with the administration of ATV/r and rifampicin occurring simultaneously. A similar simulation was generated in non‐pregnant virtual adults for comparisons. The standard regimen explored was oral 300 mg ATV and oral 100 mg RTV repeated once daily (ATV/r 300/100 mg OD), and oral 600 mg rifampicin OD. This co‐administration was studied in the virtual population during second and third trimesters of pregnancy. ATV PK at steady‐state were analyzed for each simulation. In addition, the predicted ATV *C*_trough_ at steady‐state was computed to determine the percentage of the virtual population with ATV *C*_trough_ above ATV PAIC_90_ (14 ng/mL).

ATV PK with alternative dosing regimens of ATV/r were also explored in second and third trimesters of pregnancy. The alternative dosing regimens were ATV/r 300/100 mg twice daily (BD), ATV/r 300/200 mg OD, and ATV/r 300/200 mg BD. Each alternative dosing regimen of ATV/r was simulated as being co‐administered with 600 mg rifampicin repeated OD. ATV PK at steady‐state were analyzed for each simulation and predicted ATV *C*_trough_ at steady‐state was computed to determine the percentage of the virtual population with ATV *C*_trough_ above ATV PAIC_90_ (14 ng/mL).

## RESULTS

### Pregnancy PBPK model validation with clinical PK data during pregnancy

Comparisons between the simulated PK data of oral ATV/r 300/100 mg OD and the corresponding observed PK data, from Mirochnick et al. (2011) and Conradie et al. (2011),^13^, ^14^ are shown in Table 3 and Figure S2. Calculated AAFE values were all less than twofold which was the stated acceptance threshold for this study, but ATV AUC and *C*_max_ were generally over‐predicted. In addition, comparisons of plasma concentrations at corresponding datapoints between the predicted and the sparse PK data collected from pregnant women participating in the ‘VirTUAL’ clinical trial (NCT03923231) also yielded AAFE values of 1.47 and 1.63 for ATV, and AAFE values of 1.92 and 1.52 for RTV, during the second and third trimester of pregnancy, respectively. Also, comparisons between the observed and corresponding simulated PK data of oral 600 mg rifampicin repeated once daily in the third trimester of pregnancy are shown in Table 3 with AAFE values <2. Hence, the pregnancy PBPK model was considered validated for simulating the disposition of oral ATV/r PK and rifampicin PK during pregnancy.

**TABLE 3:** Validation of pregnancy PBPK model with PK of oral ATV/r 300/100 mg once daily alone and oral 600 mg rifampicin once daily alone (simulated vs. observed).

PK parameter	Observed	Simulated	AAFE
ATV (3rd trimester)^a^			
*C*_trough_ (mg/L)	0.7	0.84	1.20
*C*_max_ (mg/L)	3.60	5.49	1.53
AUC_0‐24_ (mg.h/L)	41.9	68.2	1.63
RTV (3rd trimester)^a^			
*C*_max_ (mg/L)	0.80	0.77	1.04
AUC_0‐24_ (mg.h/L)	5.7	7.6	1.34
ATV (2nd trimester)^b^			
*C*_trough_ (mg/L)	0.66	0.76	1.14
*C*_max_ (mg/L)	3.73	5.27	1.42
AUC_0‐24_ (mg.h/L)	34.4	67.5	1.96
ATV (3rd trimester)^b^			
*C*_trough_ (mg/L)	0.67	0.75	1.12
*C*_max_ (mg/L)	3.29	5.36	1.63
AUC_0‐24_ (mg.h/L)	34.3	68.3	1.99
ATV (3rd trimester)^b^, ^c^			
*C*_trough_ (mg/L)	0.92	1.00	1.09
*C*_max_ (mg/L)	4.21	7.10	1.69
AUC_0‐24_ (mg.h/L)	46.6	90.4	1.94
RIF (3rd trimester)^d^			
*C*_max_ (mg/L)	8.40	7.65	1.10
AUC_0‐24_ (mg.h/L)	40.8	41.0	1.00

Abbreviations: AAFE, absolute average fold error; ATV, atazanavir; ATV/r, ritonavir‐boosted atazanavir; AUC_0‐24_, area under the plasma concentration time curve within 24 h; *C*_max_, maximum plasma concentration; *C*_trough_, plasma concentration at the end of the dosing interval; RTV, ritonavir; RIF, rifampicin.

^a^ Observed data reported by Mirochnick et al.^14^ as median values.

^b^ Observed data reported by Conradie et al.^13^ as geomean values.

^c^ ATV/r 400/100 mg OD.

^d^ Observed data reported by Denti et al.^15^ as median values.

### Predictions for co‐administration of ATV/r with 600 mg rifampicin once daily in pregnancy

**FIGURE 1:**
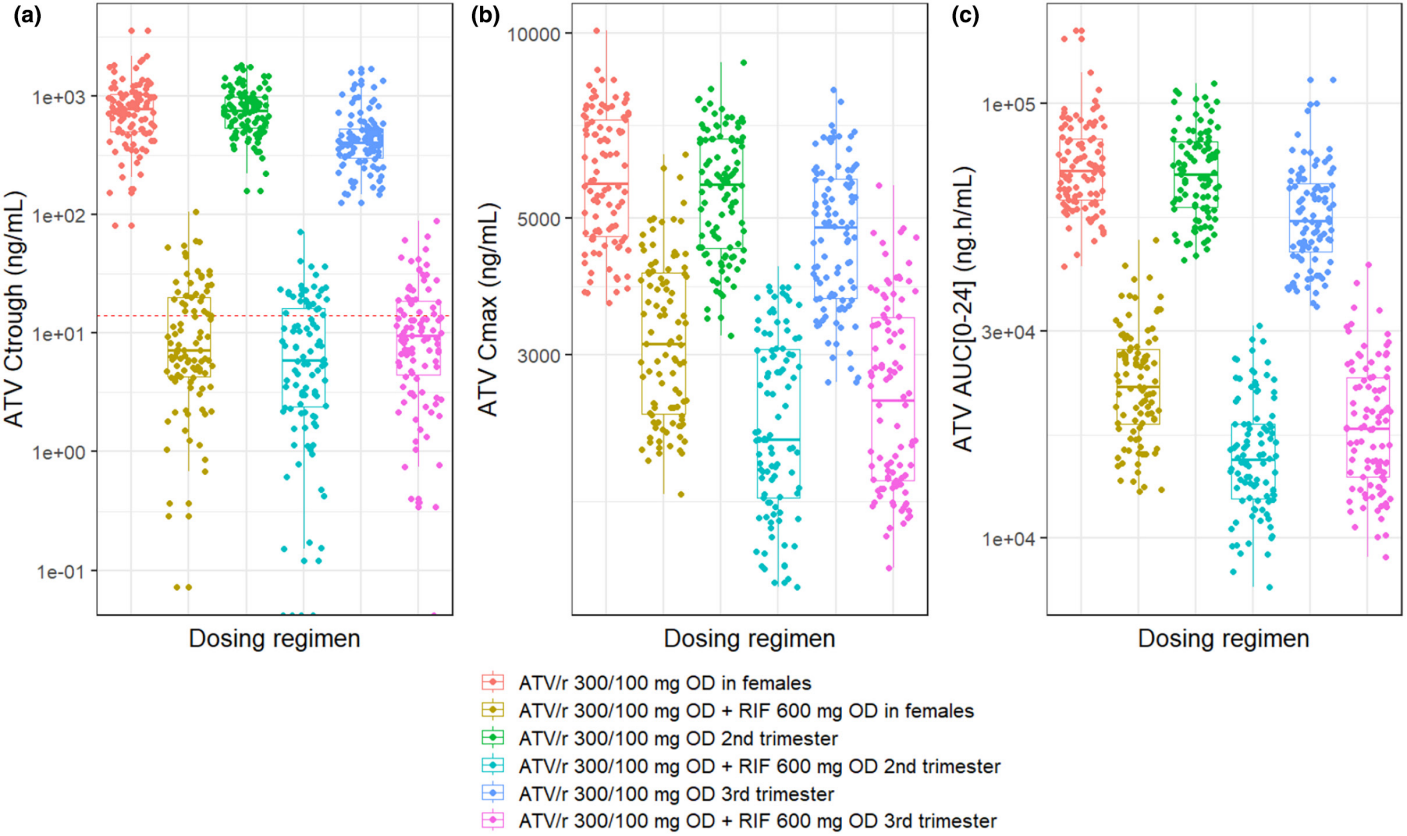
Boxplot of predicted ATV PK with ATV/r 300/100 mg in non‐pregnant adult females and when co‐administered with or without 600 mg rifampicin once daily in pregnant and non‐pregnant adult females. (a)—ATV Ct_rough_, (b)—ATV *C*_max_, (c)—AUC_0‐24_. Red dotted line in (a) is the ATV PAIC_90_ = 14 ng/mL. ATV, atazanavir; ATV/r, ritonavir‐boosted atazanavir; RIF, rifampicin.

Figure 1 shows the predicted PK of ATV at steady‐state during repeated once‐daily administration of ATV/r 300/100 mg in pregnant and non‐pregnant women with the co‐administration of rifampicin 600 mg once daily. Comparing the ATV PK with and without the addition of rifampicin, predicted geometric mean ratios (GMR) for ATV *C*_trough_, *C*_max_, and AUC_0‐24_ were 0.01, 0.55, and 0.32, respectively in non‐pregnant women indicating the predicted impact of rifampicin DDI effect in non‐pregnant women. A similar comparison in pregnant women resulted in the predicted GMR for ATV *C*_trough_, *C*_max_, and AUC_0‐24_ as 0.01, 0.40, and 0.22, respectively, for the second trimester; and as 0.01, 0.44, and 0.25, respectively, for the third trimester of pregnancy. These predictions illustrate the additive effect of pregnancy and the DDI effect of rifampicin on ATV PK in pregnant women.

The predicted effect of pregnancy alone was also computed by comparing the ATV PK in pregnancy against non‐pregnant women receiving only ATV/r without rifampicin. With this comparison, GMR for ATV *C*_trough_, *C*_max_, and AUC_0‐24_ were 1.00, 0.95, and 0.97, respectively, in the second trimester; and 0.58, 0.80, and 0.77, respectively, in the third trimester of pregnancy. Likewise, the predicted effect of pregnancy alone was also computed by comparing the ATV PK in pregnancy against non‐pregnant women when both ATV/r and rifampicin were co‐administered. With this comparison, GMR for ATV *C*_trough_, *C*_max_, and AUC_0‐24_ were 0.70, 0.72, and 0.68, respectively, in the second trimester, and 1.02, 0.80, and 0.80, respectively, in the third trimester of pregnancy. In addition, 34%, 29%, and 32% of the non‐pregnant women, pregnant women in second and third trimesters, respectively, receiving both standard doses of ATV/r (300/100 mg) and rifampicin were predicted to have ATV *C*_trough_ above 14 ng/mL.

**FIGURE 2:**
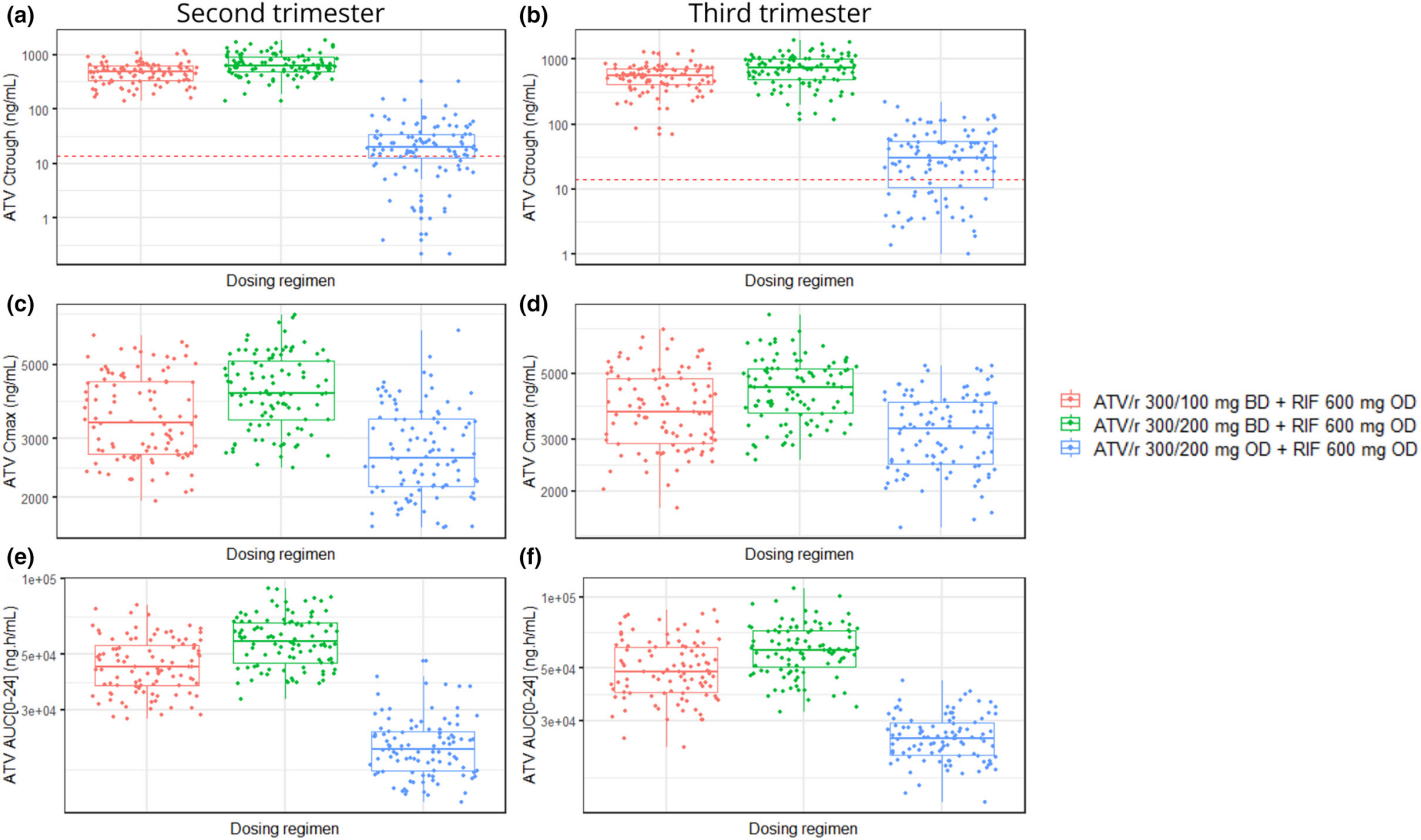
Boxplot of predicted ATV PK with multiple alternative dosing regimens of ATV/r when co‐administered with 600 mg rifampicin once daily. ATV/r dosing regimens explored include ATV/r 300/100 mg BD, ATV/r 300/200 mg BD and ATV/r 300/200 mg OD. (a, b) Predicted ATV *C*_trough_ in second and third trimester of pregnancy respectively, (c, d) Predicted ATV *C*_max_ in second and third trimester of pregnancy respectively, (e, f) Predicted AUC_0‐24_ in second and third trimester of pregnancy respectively. Red dotted line in (a & b) is the ATV PAIC_90_ = 14 ng/mL. ATV, atazanavir; ATV/r, ritonavir‐boosted atazanavir; RIF, rifampicin.

Figure 2 shows predicted ATV PK at steady‐state in second and third trimesters, respectively, of pregnancy during co‐administration of rifampicin 600 mg OD with alternative dosing regimens of ATV/r that were explored in this study. With ATV/r 300/200 mg OD co‐administered with rifampicin 600 mg OD, 71% and 73% of the pregnant population had predicted ATV *C*_trough_ above 14 ng/mL during the second and third trimesters of pregnancy, respectively.

With the twice daily dosing of ATV/r, ATV PK values were generally predicted to be slightly higher during the first 12 h after rifampicin dosing compared to the latter 12 h [data not shown]. Nonetheless, the DDI impact of rifampicin on ATV *C*_trough_ was significantly reduced with the twice daily dosing regimens of ATV/r explored. ATV *C*_trough_ was predicted to be above 14 ng/mL in all virtual patients receiving ATV/r 300/100 mg BD with rifampicin 600 mg OD. In addition, their predicted GMR for ATV *C*_trough_, *C*_max_, and AUC_0‐24_ in the second trimester of pregnancy were 0.62, 0.60, and 0.63, respectively, in comparison to the corresponding predicted ATV PK with ATV/r 300/100 mg OD in non‐pregnant women. Similarly, the lower GMR for ATV *C*_trough_, *C*_max_, and AUC_0‐24_ in the third trimester of pregnancy were 0.71, 0.64, and 0.69, respectively, in comparison to the corresponding predicted ATV PK with ATV/r 300/100 mg OD in non‐pregnant women.

ATV *C*_trough_ was also predicted to be above 14 ng/mL in all virtual pregnant patients receiving ATV/r 300/200 mg BD with rifampicin 600 mg OD. Similarly, their predicted GMR for ATV *C*_trough_, *C*_max_, and AUC_0‐24_ in the second trimester of pregnancy were 0.89, 0.71, and 0.79, respectively, in comparison to the corresponding predicted ATV PK with ATV/r 300/100 mg OD in non‐pregnant women. Also, the lower GMR for ATV *C*_trough_, *C*_max_, and AUC_0‐24_ in the third trimester of pregnancy were 0.95, 0.75, and 0.83, respectively, in comparison to the corresponding predicted ATV PK with ATV/r 300/100 mg OD in non‐pregnant women.

## DISCUSSION

In this study, we used PBPK modeling to investigate dosing strategies for ATV/r that could overcome the DDI effect with rifampicin in pregnancy. For this study, a published PBPK model^11^ with DDI modeling components which was developed for investigating DDI between ATV/r and rifampicin in adults was employed. The adult PBPK model was modified into a pregnancy PBPK model. First, the sex‐specific characteristics (e.g., organ and tissue weights and volumes) were limited to female values only.^17^ Subsequently, mathematical equations used gestational age to define changes in key biological parameters such as cardiac output and relevant enzyme activities (CYP3A4 and CYP2D6).^12^ The pregnancy PBPK model was successfully validated with relevant pregnancy clinical data for ATV/r and rifampicin.^13^, ^14^, ^15^ However, a fetal compartment was not included in the pregnancy PBPK model as ATV and rifampicin do not distribute appreciably into the fetal compartment during pregnancy.^6^, ^25^

The ATV/r dosing strategies investigated in the presence of rifampicin 600 mg once daily during pregnancy included ATV/r 300/200 mg once daily, ATV/r 300/100 mg twice daily, and ATV/r 300/200 mg twice daily. Model predictions indicate that ATV/r 300/200 mg once daily would not surmount the DDI effect of rifampicin during pregnancy as over a quarter of the pregnant population were predicted to have *C*_trough_ below 14 ng/mL. Conversely, both twice daily dosing of ATV/r (i.e., ATV/r 300/100 mg twice daily and ATV/r 300/200 mg twice daily) were predicted to sufficiently raise ATV *C*_trough_ to overcome the DDI effect of rifampicin. In addition, the boosting effect of ATV/r twice daily dosing on ATV *C*_trough_, the parameter of greatest clinical importance, was predicted to be more substantial than corresponding increases in ATV *C*_max_ and AUC_0‐24_ in pregnancy. These predictions for pregnancy agree to both predicted and observed effect of twice daily dosing of ATV/r 300/100 mg on the ATV *C*_max_ and AUC in non‐pregnant adults compared to ATV *C*_trough_.^8^, ^11^

Arguably, boosting and maintaining the plasma *C*_trough_ above a therapeutic threshold is of greatest importance in this case. Nonetheless, clinical studies investigating twice daily dosing of ATV/r 300/100 mg in the presence and absence of rifampicin in pregnancy would be needed to confirm these model predictions. Of note, the commonly cited minimum effective concentration target for ATV *C*_trough_ at 150 ng/mL has come under question. Available data suggests the protein‐adjusted 90% inhibitory concentration of ATV (14 ng/mL) might be more reliable.^8^

Lastly, the safety of this dose escalation in pregnancy warrants further safety investigations before implementation. Currently, ritonavir‐boosted PIs have been associated with adverse pregnancy outcomes such as low birth weight, small for gestational age, and miscarriage.^1^ It is unclear if dose escalation of ATV/r 300/100 mg once daily to twice daily in pregnant women concomitantly receiving standard doses of rifampicin might increase the risks of these adverse pregnancy outcomes. However, increased risks related to ATV concentrations might be unlikely with twice daily dosing of ATV/r 300/100 mg due to similar ATV exposure in the presence of DDI.

Whole‐body PBPK models for rifampicin have been previously reported in literature.^26^ However, this study used a three‐compartment PBPK model to represent rifampicin disposition. Ritonavir PBPK models without transporter activity have been earlier reported, but the absence of the transporter activity was considered a limitation.^27^, ^28^ More recent ritonavir PBPK models developed have reported including the effect of ritonavir on transporter activities,^11^, ^29^, ^30^ with some authors also accounting for intestinal transporters.^30^ In addition, PBPK models predicting very complex DDIs have been reported in literature.^11^, ^31^ Similarly, the effect of pregnancy could influence DDIs in pregnant women compared to healthy volunteers that are often recruited for clinical DDI studies. In this modeling study, we have attempted to incorporate the effect of pregnancy on both the victim and perpetrator drugs toward improving our predictions.

Limitations of the pregnancy PBPK model are similar to points earlier highlighted by Montanha et al.^11^ for the adult PBPK model. A time‐lag would be expected before equilibrium is reached because drugs move across membranes via passive diffusion. This time‐lag could affect the plasma drug concentration at a given time though it is less likely to affect the total exposure of the drug (AUC).^11^ Likewise, there are challenges about determining the mechanisms of membrane drug transporters in vitro, but these transporters have a relevant impact on tissue distribution especially for ATV distribution.^11^ Another limitation of the model is the non‐representation of the DDI effect on efflux transporter (P‐gp) in the intestine.

Also, the use of a simple compartment model for the disposition of rifampicin means the validated adult model cannot be easily extended to simulate for an alternative population. For instance, where clinical data on rifampicin's apparent drug clearance and absorption rate constants within the desired population are not available, the simple PK model cannot be parameterized for that population. With a more mechanistic model, changes in the biological system parameters could adapt the model to predict rifampicin PK more easily for a different population. Nonetheless, the model would be considered fit‐for‐purpose in this study as there were rifampicin PK data during pregnancy to build and also validate the pregnancy model for rifampicin.

The possibility of safely overcoming the DDI of rifampicin on ATV PK in pregnancy by escalating the dosing of ATV/r 300/100 mg once daily to twice daily is very important as this would increase available treatment options for pregnant women that might need to co‐treat HIV and TB. The model predictions suggest dose escalation of ATV/r once daily to twice daily in pregnancy might overcome the DDI impact of rifampicin on ATV *C*_trough_ which is the most clinically‐relevant PK parameter in this case. Further clinical studies in pregnant women are still warranted. This study reiterates the usefulness of PBPK modeling to investigate various complex clinical scenarios within complex populations.

## AUTHOR CONTRIBUTIONS

All authors wrote the manuscript. C.W., M.S., P.D., and S.A. designed the research. S.A. performed research and analyzed the data. M.S. contributed analytical tools.

## FUNDING INFORMATION

This project is part of the EDCTP2 program supported by the European Union (grant number RIA2016MC‐1606‐VirTUAL). CW is funded by Wellcome Trust (222075_Z_20_Z). SA was supported by the Duncan Norman Charitable Trust.

## CONFLICT OF INTEREST STATEMENT

S.A., C.W., R.N., H.M., C.O., and P.D.—No conflicts of interest to declare. M.M. is currently a paid employee of Bristol Myers Squibb. M.S. is currently employed by esqLABS. As an Associate Editor for *CPT: Pharmacometrics & Systems Pharmacology*, Paolo Denti was not involved in the review or decision process for this paper.

## Supporting information


Data S1:
