# Supplementary material for: Physiologically based pharmacokinetic modeling of drug–drug interactions between ritonavir‐boosted atazanavir and rifampicin in pregnancy
Source: CPT Pharmacometrics Syst Pharmacol. 2024 Nov 8;13(11):1967–77. doi: 10.1002/psp4.13268 (PMC11578135; doi:10.1002/psp4.13268)
Supplement: Supplementary file 1 — Data S1: [file PSP4-13-1967-s001.docx]

# **Supplementary Information Titles**

Table S1: Drug-specific parameters for ATV, RTV and RIF implemented in the model as reported by Montanha et al (2022)^1^

| Drug parameter | ATV | RTV | RIF |
| --- | --- | --- | --- |
| pKa | 13 | 2.8 | - |
| B:P | 0.75 | 0.58 | - |
| Log P_O:W_ | 2.9 | 3.3 | - |
| F_u,p_ | 0.14 | 0.02 | - |
| Solubility (mg/mL) | 4.5 | 0.09 | 1.4 |
| Caco2 P_app_ x 10^-6^ (cm/s) | 5.30 | 3.9 | - |
| K_a_ (h^-1^) | - | - | 1.15 |
| CYP3A4 CL_int,liver_ (μL/min/pmol) | 0.3 | 21.3 | - |
| CYP2D6 CL_int,liver_ (μL/min/pmol) | - | 0.93 | - |
| CYP3A4 CL_int,gut_ (μL/min/pmol) | 0.3 | 17 | - |
| Cl_int,up,pas_ (μL/min/million cells) | 134 | - | - |
| Cl_int,eff_ (μL/min/million cells) | 7 | - | - |
| Cl_int,up,act_ (μL/min/million cells) | 40 | - | - |
| Apparent clearance (L/h) | - | - | 19.2 |
| V_ss_ (L) | - | - | 53.2 |

ATV – atazanavir; RTV – ritonavir; RIF – rifampicin; pKa – acid dissociation constant; B:P – blood-to-plasma drug partition coefficient; Log P_o:w_ – drug partition coefficient between n-octanol and water; F_u,p_ – fraction of unbound drug in the plasma; P_app_ – apparent drug permeability coefficient with Caco2 cells; CL_int,liver_ – intrinsic drug clearance in the liver by the CYP enzyme; CL_int,gut_ – intrinsic drug clearance in the gut by the CYP enzyme; CYP – cytochrome P450; K_a_ – drug absorption constant; Cl_int,up,pas_ – intrinsic passive uptake clearance; Cl_int,eff_ – intrinsic efflux clearance; Cl_int,up,act_ – intrinsic active uptake clearance; and V_ss_ – volume of distribution at steady state.

Table S2: Induction parameters for RTV and RIF as reported by Montanha et al (2022)^1^.

| Drug parameter | RTV | | RIF | |
| --- | --- | --- | --- | --- |
|  | E_max_ | EC_50_ (µM) | E_max_ | EC_50_ (µM) |
| CYP3A4 | 13.4 | 0.44 | 12.3 | 0.85 |
| Uptake transporter | - | - | 5 | 0.15 |
| Efflux transporter | - | - | 2 | 0.15 |

RTV – ritonavir; RIF – rifampicin; E_max_ - maximum induction, EC_50_ - concentration of the inducer which results in 50% of the maximum induction; and CYP – cytochrome P450.

Table S3: Inhibition parameters for RTV and RIF as reported by Montanha et al (2022)^1^.

| Drug parameter | RTV | | RIF |
| --- | --- | --- | --- |
|  | K_inact_ | K_i_ (µM) | K_i_ (µM) |
| CYP3A4 | 4.68 | 0.07 | NA |
| Uptake transporter | - | 0.78 | 0.9 |
| Efflux transporter | - | 0.3 | NA |

RTV – ritonavir; RIF – rifampicin; K_inact_ –maximal enzyme inactivation rate constant; K_i_ –concentration of the inhibitor resulting in 50% of the maximum inhibition; and CYP – cytochrome P450.

Table S4: Simulated weight distribution of organ/tissues in non-pregnant women

| Organ/tissue in kg | Mean (CV) |
| --- | --- |
| Blood | 4.29 (17) |
| Bones | 3.75 (17) |
| Brain | 1.31 (7) |
| Gonads | 0.09 (16) |
| Heart | 0.22 (16) |
| Intestines | 0.83 (17) |
| Kidneys | 0.25 (19) |
| Liver | 1.30 (14) |
| Lungs | 0.34 (22) |
| Muscle | 21.90 (55) |
| Pancreas | 0.10 (17) |
| Remaining organs | 2.32 (13) |
| Skin | 3.03 (48) |
| Spleen | 0.12 (20) |
| Stomach | 0.12 (17) |

Table S5: Simulated organ blood flow in non-pregnant and pregnant women.

| Organ blood-flow in L/h | Non-pregnant women  Mean (CV) | Second trimester  Mean (CV) | Third trimester  Mean (CV) |
| --- | --- | --- | --- |
| Adipose | 20.0 (22) | 29.9 (2.3) | 31.1 (0.28) |
| Bone | 16.2 (22) | 14.2 (2.3) | 14.8 (0.28) |
| Brain | 42.3 (22) | 33.7 (2.3) | 35.1 (0.28) |
| Gonads | 3.8 (22) | 45.9 (2.3) | 47.9 (0.28) |
| Gut | 19.2 (22) | 14.5 (2.3) | 15.2 (0.28) |
| Hepatic artery | 46.2 (22) | 18.4 (2.3) | 19.2 (0.28) |
| Kidneys | 67.4 (22) | 63.5 (2.3) | 66.3 (0.28) |
| Lungs | 9.6 (22) | 6.9 (2.4) | 7.2 (0.28) |
| Muscles | 73.1 (22) | 33.7 (2.3) | 35.1 (0.28) |
| Pancreas | 19.2 (22) | 14.5 (2.3) | 15.2 (0.28) |
| Skin | 23.1 (22) | 33.3 (2.3) | 34.7 (0.28) |
| Spleen | 19.2 (22) | 14.5 (2.3) | 15.2 (0.28) |
| Stomach | 19.2 (22) | 14.5 (2.3) | 15.2 (0.28) |

Second trimester – week 14-26; Third trimester- week 29-40


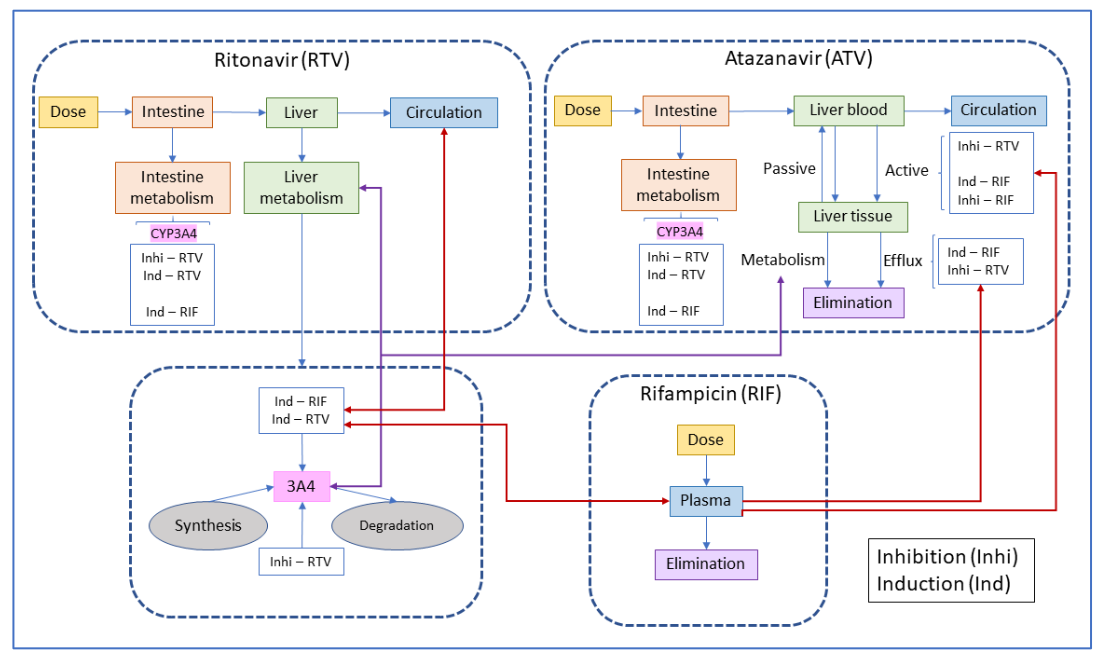


Figure S1: Schematic representation of the PBPK models used for modelling the disposition of the study drugs obtained from Montanha et al 2022 ^1^.


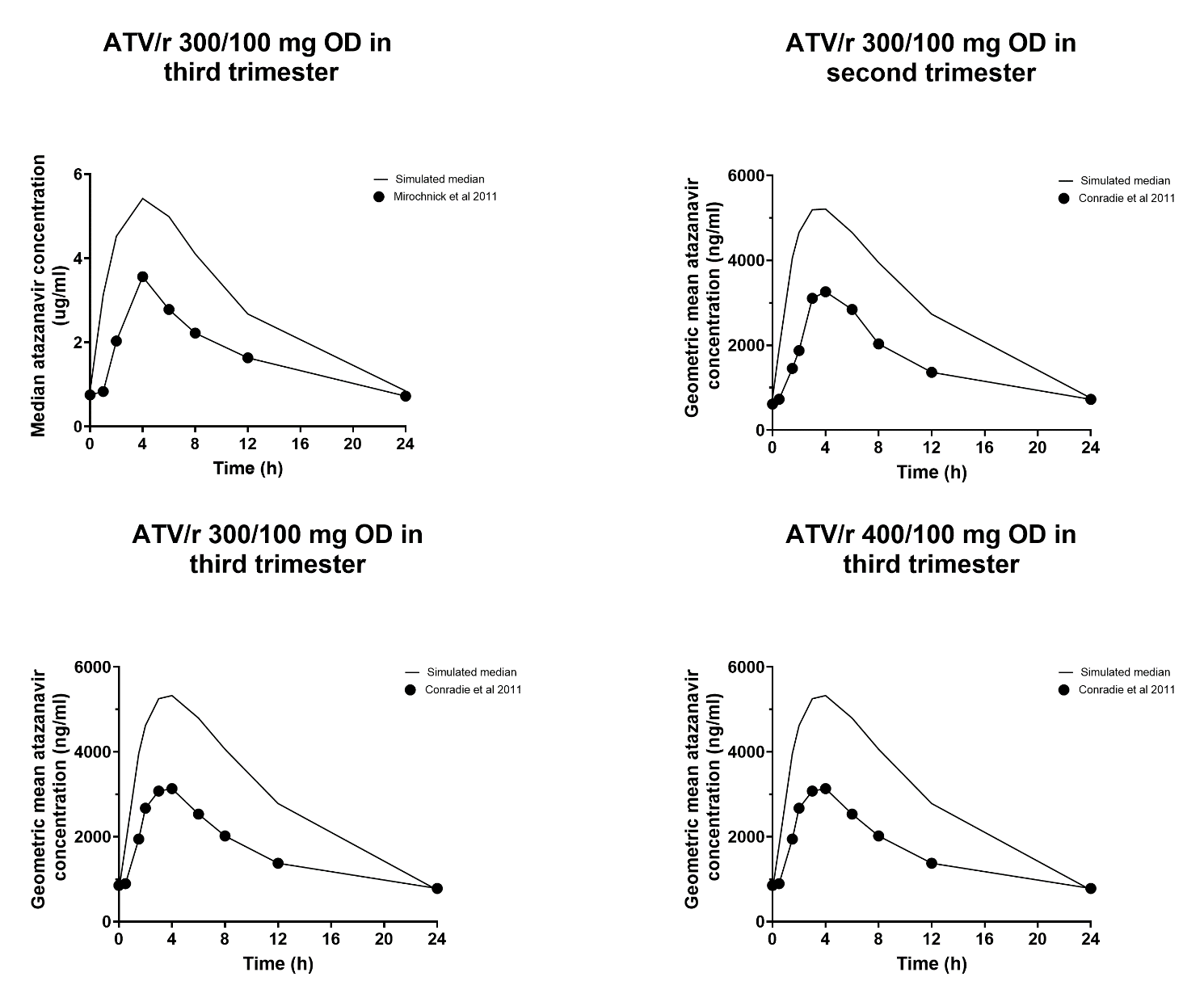


Figure S2: Observed vs simulated PK of ATV/r during pregnancy. Observed clinical data obtained by digitising relevant plasma concentration-time profiles reported by Mirochnick et al (2011) ^2^ and Conradie et al (2011) ^3^.

Reference

1. Montanha MC*, et al.* Predicting Drug–Drug Interactions between Rifampicin and Ritonavir-Boosted Atazanavir Using PBPK Modelling. *Clinical Pharmacokinetics* **61** 375-386. (2022)

2. Mirochnick M*, et al.* Atazanavir pharmacokinetics with and without tenofovir during pregnancy. *Journal of acquired immune deficiency syndromes (1999)* **56** 412. (2011)

3. Conradie F*, et al.* Safety and exposure of once-daily ritonavir-boosted atazanavir in HIV-infected pregnant women. *HIV Medicine* **12** 570-579. (2011)
